# Trophic interactions between larger crocodylians and giant tortoises on Aldabra Atoll, Western Indian Ocean, during the Late Pleistocene

**DOI:** 10.1098/rsos.171800

**Published:** 2018-01-24

**Authors:** Torsten M. Scheyer, Massimo Delfino, Nicole Klein, Nancy Bunbury, Frauke Fleischer-Dogley, Dennis M. Hansen

**Affiliations:** 1 University of Zurich, Palaeontological Institute and Museum, Karl Schmid-Strasse 4, 8006 Zurich, Switzerland; 2 Dipartimento di Scienze della Terra, Università di Torino, Via Valperga Caluso 35, I-10125 Torino, Italy; 3 Institut Català de Paleontologia Miquel Crusafont, Universitat Autònoma de Barcelona, Edifici ICTA-ICP, Carrer de les Columnes s/n, Campus de la UAB, Cerdanyola del Vallès, Barcelona 08193, Spain; 4 Steinmann Institut für Geologie, Paläontologie und Mineralogie, Universität Bonn, Nussallee 8, 53115 Bonn, Germany; 5 Seychelles Islands Foundation, PO Box 853, Victoria, Mahé, Seychelles; 6 Zoological Museum and the Department of Evolutionary Biology and Environmental Studies, University of Zurich, Winterthurerstrasse 190, 8057 Zurich, Switzerland

**Keywords:** Seychelles, predator–prey interaction, scavenging, Testudines, *Aldabrachelys*, *Aldabrachampsus*

## Abstract

Today, the UNESCO World Heritage Site of Aldabra Atoll is home to about 100 000 giant tortoises, *Aldabrachelys gigantea*, whose fossil record goes back to the Late Pleistocene. New Late Pleistocene fossils (age *ca*. 90–125 000 years) from the atoll revealed some appendicular bones and numerous shell fragments of giant tortoises and cranial and postcranial elements of crocodylians. Several tortoise bones show circular holes, pits and scratch marks that are interpreted as bite marks of crocodylians. The presence of a Late Pleistocene crocodylian species, *Aldabrachampsus dilophus*, has been known for some time, but the recently found crocodylian remains presented herein are distinctly larger than those previously described. This indicates the presence of at least some larger crocodylians, either of the same or of a different species, on the atoll. These larger crocodylians, likely the apex predators in the Aldabra ecosystem at the time, were well capable of inflicting damage on even very large giant tortoises. We thus propose an extinct predator–prey interaction between crocodylians and giant tortoises during the Late Pleistocene, when both groups were living sympatrically on Aldabra, and we discuss scenarios for the crocodylians directly attacking the tortoises or scavenging on recently deceased animals.

## 1. Background

The identification of species interactions in the form of predation is fundamental to understand ecosystem complexity and community structure [1], but evidence of predator–prey interactions is rarely preserved in the fossil record [2]. Known cases are either related to rare direct evidence, such as finding interlocked and associated skeletons (e.g. [3,4]) or isolated teeth embedded in or around bones [5,6], and, more frequently, stomach contents and feeding traces (e.g. [7–10]).

Predation specifically on Testudines (turtles and tortoises) has been documented in the fossil record and manifold predators have been identified, including fishes, reptiles and mammals (e.g. [11]). Besides tortoise remains with cut marks and tooth marks, or those found associated with tools left by humans (among others, [12–15]), Late Cretaceous protostegid turtles were reported to have been bitten by sharks [16], an undetermined Late Cretaceous marine turtle has been ingested by a large lamniform shark [17], and mosasaurs are thought to be the cause of circular depression bite marks in the shell bones of Cretaceous sea turtles such as *Protostega gigas* [18–20]. Badger predation on a European pond turtle in the Holocene was reported, where the attack occurred from the anterior opening of the turtle shell, leaving irregular bite marks [2]. Protostegid turtle remains also showed up as gut content in an Early Cretaceous ichthyosaur (*Platypterygius longmani*) from Australia [21]. Finally, compression punctures and tapering scratches have been identified as bite marks in a number of fossil turtles from the Eocene of North America, caused by either mammals (carnivorans, ‘creodonts’ and ‘condylarth’ mammals) or crocodylians such as *Borealosuchus* and *Allognathosuchus*, among others [22].

Indeed, crocodylian predation on turtles (only a few are given here; see [23] for more documented examples) would be expected to be common as these two groups often occur in the same habitat [10]. Early Campanian protostegid turtles (*Chelospargis advena*) and marine pleurodire turtles (e.g. *Chedighaii barberi*) were likely bitten by the giant alligatorid *Deinosuchus* [24], leaving round- or teardrop-shaped crater-like collapse structures or circular holes in the shell bones. Possible crocodylian biting traces on bones (including turtle shell bones) that do not pierce the bones were described as the ichnofossil *Machichnus bohemicus* from the Miocene Ahníkov site in Czech Republic [25,26]. Similar marks were also found on an early Palaeocene turtle shell fragment from Denmark [27]. Predation on a Late Cretaceous bothremydid turtle (*Foxemys*) in Iharkút, Hungary, was probably done by a large *Allodaposuchus*-like crocodyliform [10]. The authors point out, however, that an adult specimen of *Foxemys* would likely have been too large to be directly attacked by an eusuchian crocodyliform, and that it would thus be more reasonable to assume the crocodyliform was scavenging on an already dead individual.

To positively identify potential predation on fossils, comparison with extant evidence is necessary. Binford [28] developed a classification scheme for bite marks and feeding traces which still serves as basis for many subsequent studies (and is also followed herein), such as those of Drumheller & Brochu [23,29], which also reported a broad spectrum of extant animals that can act as turtle predators. According to contact area, penetration depth and movement criteria of the teeth, feeding traces on bones can be classified as pits, punctures, scores and furrows [28], with the former two categories lacking lateral movement of the marking tooth (also see [23] for further discussion).

Tiger sharks bite out large parts of shell and soft tissues of marine turtles [30–32] (see also [33] for further discussion), whereas attacks on leatherback turtles by crocodiles (likely by *Crocodylus porosus* rather than the smaller *C. novaeguineae*, though not explicitly stated therein) included decapitation of one individual in shallow waters close to a nesting beach site near Piguwa, Papua New Guinea [34]. A range of terrestrial mammals, including racoons, bears, coyotes, badgers and jaguars, prey on turtles and tortoises, both on hatchlings and on larger individuals [35–37]. Predation on hatchlings or small juvenile turtles, as well as on tortoises, is also documented for birds [38,39] and crabs [40]. These predators usually lack the necessary forces to puncture older individuals' shells with their beaks and claws [33,41]. Predatory birds are known to overcome this, by dropping turtles/tortoises from great heights to crack them open [42], and at least the coconut crab, *Birgus latro*, has strong enough chelae to sever limbs and crush bones of larger-sized turtles and tortoises [43]. Predation by dwarf caimans on red-eared sliders [44] is evidenced by scratches and puncture marks, and in a documented case the turtle shell was crushed with the bridge region in a vertical orientation. Similar cases of predation by *Alligator mississippiensis* [45] have been observed, but the shells can also be crushed when held in horizontal position. For that, hard-shelled prey items such as turtles are often manipulated to the posterior part of the snout by crocodylians either inertially or gravitationally [46–48]. Despite attempts to classify some extinct crocodylians as specifically ‘cheloniphagous’ (turtle-eating) taxa (e.g. [49–53]), globular teeth are not necessarily an indicator that turtles are part of the diet [54] (see [10,45] for further discussion). Predation marks on the extinct Bahamas giant tortoise, *Chelonoidis alburyorum*, were suggested to have been caused by the Cuban crocodile, *Crocodylus rhombifer* [55]. This crocodile is usually 2–2.5 m in body length, but can reach up to 3.5 m, with a recorded maximum size of 4.9 m [56,57]. The crocodile-inflicted bite marks in the form of sub-circular or ovoid holes on the shell were in some cases healed over, providing evidence that this giant tortoise survived the initial attack.

Modern giant tortoises in the Seychelles and those of the Galapagos Islands are much larger than the giant tortoise from the Bahamas, and they currently do not share habitats with crocodylians. In the recent past, however, at least on the Seychelles and on Madagascar, giant tortoises and crocodylians seem to have shared habitats and thus probably interacted with each other [58–61]. Little is known, on the other hand, about the palaeobiogeographic history and body size evolution of the Aldabran crocodylians and their impact on the ancient island ecosystem. We here thus report on novel fossil remains from Aldabra, providing the first evidence of a trophic interaction in the form of predation of larger crocodylians on the giant tortoises during the Late Pleistocene.

## 2. Material and methods

### 2.1. Fossils

**Figure 1.**
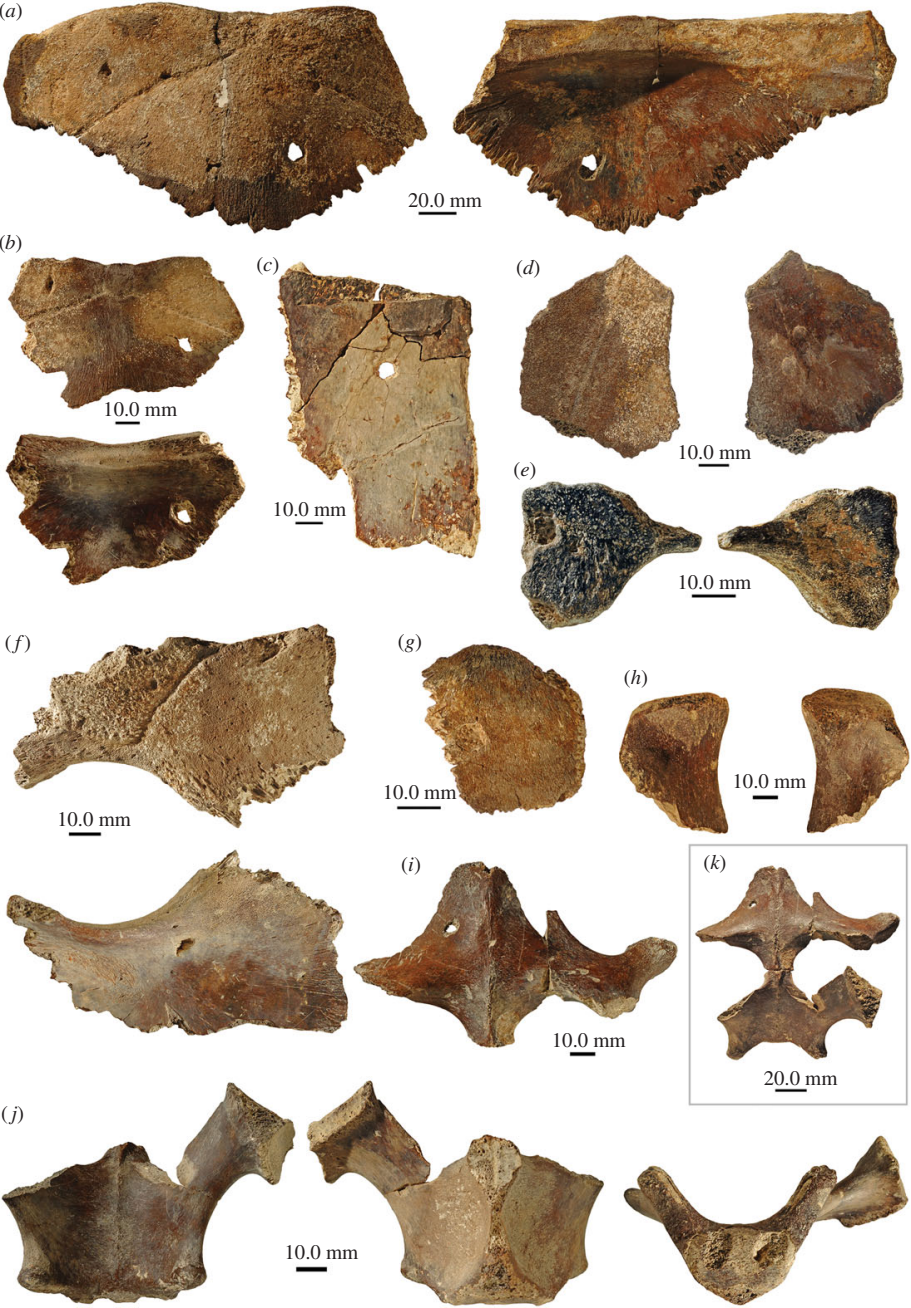
Overview of new giant tortoise material from the Late Pleistocene of Aldabra Atoll. (*a*) Large nuchal (SNHM 1448/17) still sutured to first left peripheral in dorsal and ventral view; (*b*) small nuchal (SNHM 1449/17) in dorsal and ventral view, note cervical scute in both nuchals; (*c*) larger costal fragment (SNHM 1450/17) in ventral view; (*d*) a smaller costal fragment (SNHM 1451/17) with sulcus in dorsal and ventral view; (*e*) smaller hyo- or hypoplastron fragment (SNHM 1452/17) in ventral and dorsal view; (*f*) larger hyo- or hypoplastron fragment (SNHM 1453/17) in ventral and dorsal view; (*g*) small shell fragment (SNHM 1454/17) which might also pertain to a costal in purported dorsal view; (*h–k*) associated pelvic girdle elements; (*h*) distal part of an ilium in lateral and medial view (SNHM 1455/17); (*i*) fused pubes in angled anterodorsal view (SNHM 1456/17); (*j*) fused ischia in angled posterodorsal, angled posteroventral and posterior view (SNHM 1457/17); (*k*) fused pubes and ischia in natural articulated position in dorsal view.

Recent surface collection by a research team led by one of us (DH) in the surrounding area of a pond on Grande Terre Island on Aldabra yielded over 180 individual fossilized bones, most coming from crocodylians and the shells of giant tortoises. Several tortoise bones carry conspicuous sub-circular holes completely piercing the elements or pits that did not penetrate the bone, and other marks on the bone surface that are identifiable as feeding traces. The giant tortoise remains studied herein (figure 1) include one large nuchal with first left peripheral attached (SNHM 1448/17), one small nuchal (SNHM 1449/17), a larger costal fragment (SNHM 1450/17), a smaller costal fragment with sulcus (SNHM 1451/17), a smaller (SNHM 1452/17) and a larger (SNHM 1453/17) hyo- or hypoplastron fragment, one small shell fragment (SNHM 1454/17), which might also pertain to a costal and a pelvic girdle consisting of the distal part of a right ilium (SNHM 1455/17) and associated fused pubes (SNHM 1456/17) and fused ischia (SNHM 1457/17).

**Figure 2.**
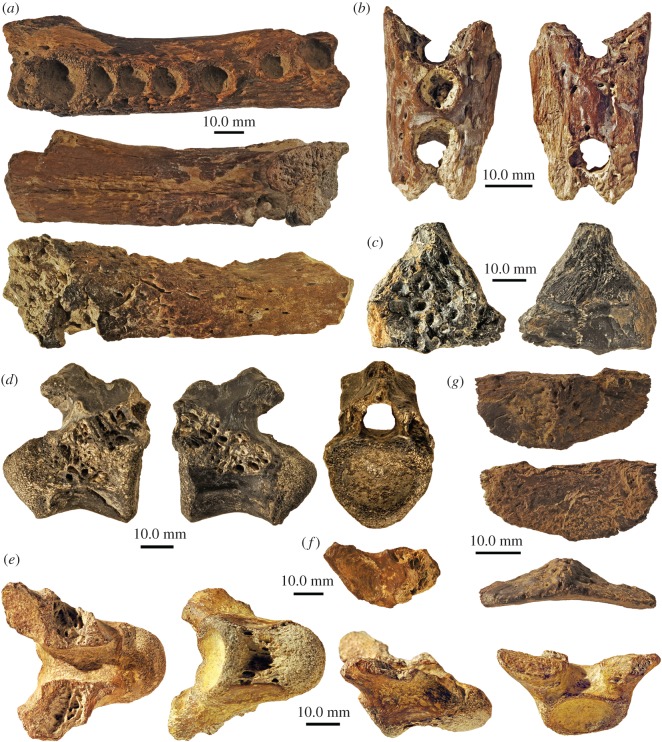
Overview of new crocodylian material from the Late Pleistocene of Aldabra Atoll. (*a*) Larger left dentary fragment (SNHM 1458/17) with alveoli d3–d8 preserved in dorsal, medial and lateral view; (*b*) small left dentary fragment (SNHM 1459/17) with alveoli d4–d6 preserved in dorsal and ventral view; (*c*) skull roof fragment (SNHM 1460/17) consisting mainly of the left postorbital and partial frontal and parietal fragments in dorsal and ventral view; (*d*) dorsal procoelous vertebra (SNHM 1461/17) with neural arch preserving the postzygapophyses in left lateral, right lateral and anterior view; (*e*) strongly eroded vertebral centrum still preserving the prezygapophyses (SNHM 1462/17) in dorsal, ventral, right lateral and anterior view; (*f*) isolated left prezygapophysis (SNHM 1463/17) in dorsal view; (*g*) posterior half of osteoderm (SNHM 1464/17) in dorsal, ventral and posterior view.

The crocodylian remains studied herein (figure 2) include one large left dentary fragment with alveoli d3–d8 preserved (SNHM 1458/17), a small left dentary fragment with alveoli d4–d6 preserved (SNHM 1459/17), a skull roof fragment consisting mainly of the left postorbital sutured to small parts of the frontal and parietal (SNHM 1460/17), one dorsal procoelous vertebra with neural arch showing only postzygapophyses (SNHM 1461/17), one strongly eroded procoelous vertebral centrum still preserving the prezygapophyses (SNHM 1462/17), one isolated left prezygapophysis (SNHM 1463/17) and the posterior half of an osteoderm (SNHM 1464/17).

Some of the crocodylian material belongs to an individual or individuals that are considerably larger than has been previously reported for the only described crocodylian from Aldabra, *Aldabrachampsus dilophus* Brochu, 2006 [62]. The possibility remains that the new material does not belong to *A. dilophus*, but could represent a larger-sized species, e.g. osteolaemines close to *Voay robustus* (note that the postorbital region in *V. robustus* shows peculiar tuberosities not present in the new sample [63]) or *Crocodylus niloticus*, instead (see [62]).

All described material derives from calcarenitic sediments of Late Pleistocene age, the so-called Aldabra Limestone with an age of 90 000–125 000 years [64], surrounding a partially dried-out pond on Grande Terre Island (coordinates S09.42759, E046.51408). All specimens are stored in the Seychelles Natural History Museum (SNHM). The bones were cleaned and prepared with a set of pneumatic scribe tools and measurements were taken with an iGaging digital caliper. Photographs were taken with a Nikon D2X camera and Nikon AF Nikkor 35–70 mm lens and resulting images were processed with Adobe Creative Suite CS6. A summary of individual measurements has been compiled into tables 1 and 2.

**Table 1.** Measurements of fossilised crocodylian remains (in mm, rounded to the first decimal place).

crocodylian remains	
larger left dentary fragment (SNHM 1458/17)	
maximum length	117.5 as preserved
anterior maximum width	37.3
posterior maximum width	26.5
maximum height at fourth dentary alveolus	36.6
maximum height at ninth dentary alveolus	30.1
symphysial scar	29.8 as preserved
width of dentary alveolus 3	6.1
width of dentary alveolus 4	15.3
width of dentary alveolus 5	9.4
width of dentary alveolus 6	9.7
width of dentary alveolus 7	9.7
width of dentary alveolus 8	8.7
width of dentary alveolus 9	8.2
width of dentary alveolus 10	11.7
thickness of bone wall separating dentary alveoli 7 and 8	6.2
thickness of bone wall separating dentary alveoli 8 and 9	11.2
thickness of bone wall separating dentary alveoli 9 and 10	4.9
smaller left dentary fragment (SNHM 1459/17)	
maximum length	41.8 as preserved
maximum width	25.3
maximum thickness	18.1 as preserved
width of dentary alveolus 4	9.3
width of dentary alveolus 5	7.9
width of dentary alveolus 6	8.2
skull roof fragment (SNHM 1460/17)	
maximum length	39.1
maximum width	40.1
maximum thickness (excluding ventral postorbital pillar)	12.3
dorsal procoelous vertebra (SNHM 1461/17)	
maximum length of centrum	42.7
maximum width of centrum (anterior border)	31.1
maximum height of centrum (anterior border, to floor of central canal)	27.6
length of neural arch	33.5 as preserved
height of neural arch	21.6 as preserved
width of neural arch (at postzygapophyses)	22.8
maximum diameter of neural canal	9.7
strongly eroded vertebra (SNHM 1462/17)	
maximum length of centrum	36.5
maximum width of centrum (anterior border)	26.3
maximum height of centrum (anterior border, to floor of central canal)	16.2
width of neural arch (at prezygapophyses)	46.7 (as preserved)
maximum diameter of neural canal	9.1
length of prezygapophyseal articulation facet	21
width of prezygapophyseal articulation facet	11.7
isolated left prezygapophysis (SNHM 1463/17)	
length of prezygapophyseal articulation facet	23.1
width of prezygapophyseal articulation facet	16.6
osteoderm fragment (SNHM 1464/17)	
maximum length	17.9 as preserved
maximum width	39.8
maximum height at median keel	9.3

**Table 2.** Measurements of fossilised giant tortoise remains (in mm, rounded to the first decimal place).

giant tortoise remains	
larger nuchal (SNHM 1448/17)	
maximum width	194.2
maximum length	127.9
maximum thickness of anterior border	26.4
maximum width of cervical scute	32.9
maximum length of vertebral sulcus (left)	103.1
posterior median thickness of nuchal	6.6
diameter of puncture (completely penetrating plate)	9.7
smaller nuchal (SNHM 1449/17)	
maximum width	97.9 as preserved (complete width estimated *ca*. 107)
maximum length	62.9 as preserved
maximum thickness of anterior border	11.9
maximum width of cervical scute	14.7
maximum length of vertebral sulcus (left)	47.3
posterior median thickness of nuchal	3.8
diameter of bisected mark (completely penetrating plate)	6.9 × 4.8
larger costal (SNHM 1450/17)	
maximum length	97.9 as preserved
maximum width	70.3 proximally
maximum thickness	5.4
diameter of puncture hole	6.6
smaller costal (SNHM 1451/17)	
maximum length	64.5 as preserved
maximum width	61.5 as preserved
maximum thickness	6.6
smaller hyo- or hypoplastron (SNHM 1452/17)	
maximum length (of flat part)	33.3
maximum width (of flat part)	31.1
diameter of pit	7.5
larger hyo- or hypoplastron (SNHM 1453/17)	
maximum length (of flat part)	61.2
maximum width (of flat part)	86.7
diameter of bisected mark	7.4 × 4.6
small shell fragment–costal fragment? (SNHM 1454/17)	
maximum length	39.8
maximum width	37.4
maximum thickness	3.7
maximum diameter of pit	7.7
distal right ilium fragment (SNHM 1455/17)	
antero-posterior length	49.1
dorsoventral length	61.4 as preserved
fused pubes (SNHM 1456/17)	
midline length	77.2 as preserved
width of right pubis measured from midline	86.9
fused ischia (SNHM 1457/17)	
midline length	57.7
width of right ischium measured from midline	61.6
maximum length of scores on posterior bone surface	12.7
maximum width of scores on posterior bone surface	8.8

### 2.2. Institutional abbreviations

BMNH, Natural History Museum, London, UK; SNHM**,** Seychelles Natural History Museum, Victoria, Mahé Island, Seychelles; ZM, Zoological Museum, University of Zurich.

## 3. Results

The newly collected crocodylian material includes three skull and mandible fragments, and postcranial remains (three vertebral remains and an osteoderm fragment). Based on the lack of diagnostic features, the fossils were identified only as Crocodyloidea indet., based on the diastema after the eighth dentary alveolus (see also [65]). Compared to extant *C. niloticus* [66,67], both the size of the dentary fragments and vertebrae ( table 1), as well as the isolated left prezygapophysis, which shows 13 incremental growth marks on the articular facet (figure 2*f*), indicate that the bones were derived from sexually mature individual(s). This assessment is supported by the complete closure of the neurocentral sutures between the vertebral centra and arches [68].

**Figure 3.**
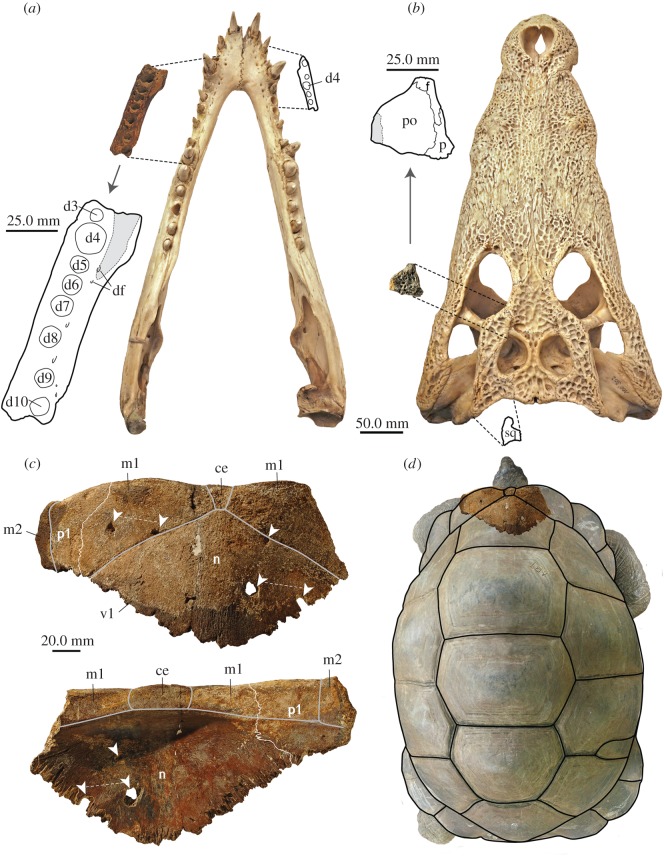
Size comparison of crocodylian and giant tortoise remains. (*a*) Image and interpretative drawing of larger left dentary fragment (SNHM 1458/17; broken bone surface area indicated by grey patch) scaled and fitted to lower jaw of extant *C. niloticus* (ZM 100.302). In addition, the outline of right dentary fragment of *Aldabrachampsus dilophus* (holotype BMNH R8795; see [62]) has been added for comparison; (*b*) image and interpretative drawing of skull roof fragment (SNHM 1460/17) consisting of the postorbital (broken bone surface area indicated by grey patch), and frontal and parietal fragments scaled and fitted to skull of extant *C. niloticus* (ZM 100.302). For comparison, the outline of the right squamosal of *Albadrachampsus dilophus* (holotype BMNH R8795; see [62]) is shown; (*c*) dorsal and ventral sides of the larger nuchal (SNHM 1448/17) with interpretative drawings of sutures and scute sulci superimposed. Note equidistance of some of the feeding traces (marked by white arrowheads connected by thin white stippled line), which might indicate repeated bites of the same crocodylian jaw portion; (*d*) larger nuchal (SNHM 1448/17) scaled to fit a large male *A. gigantea* with a head width of 95.1 mm and a CCL of 114.4 cm. This specimen has a cervical scute width of about 30 mm, comparable to the maximum width of the same element in the fossil. ce, cervical scute; m1–2, marginal scute 1–2; d3–d10, dentary alveolus 1–10; df, dental foramina; f, frontal; n, nuchal; p, parietal; p1, first peripheral; po, postorbital, sq, squamosal, v1, first vertebral scute.

The newly collected tortoise material included mostly shell bones and fragments (table 2), and also a well-preserved associated pelvic girdle. Both nuchals carry imprints of a cervical scute, and are overall morphologically indistinguishable from modern *Aldabrachelys gigantea* (Schweigger, 1812) [69]. This was already noted on earlier collections of Pleistocene tortoise material from Aldabra [70]. Comparison of the large nuchal with shells of extant *A. gigantea* (figure 3) indicates an overall size of 100–120 cm curved carapace length (CCL) of the complete fossil shell. Notably, this size is considerably larger than those of *A. gigantea* living on eastern Grande Terre today. While tortoises living on the north-western parts of the atoll (Picard and Malabar islands) can grow to sizes greater than 1 m in straight carapace length, the ones found across most of Grande Terre rarely exceed 50–60 cm (based on third scute lengths reported in Turnball *et al.* [71]). The reason for these distinct morphotypes is unknown, but suggested causes include density-dependent competition and the availability of different vegetation types [71].

**Figure 4.**
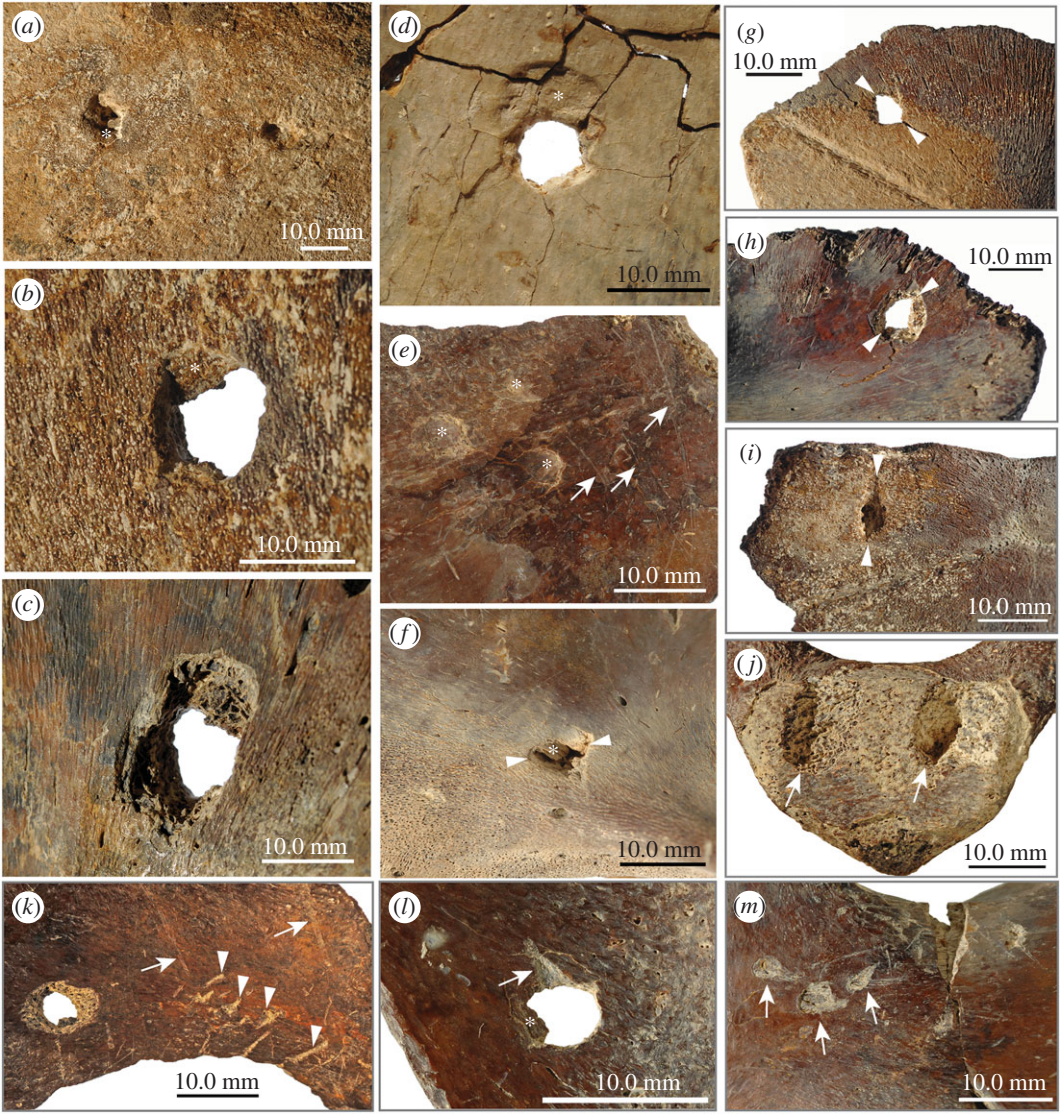
Close-up views of crocodylian feeding traces on giant tortoise bones. (*a*) Dorsal surface of larger nuchal (SNHM 1448/17): one puncture with depressed fractures (white asterisk) and one pit; (*b,c*) dorsal and ventral bone surface of larger nuchal (SNHM 1448/17): large circular puncture with depressed fractures (white asterisk), note loss of internal cortical bone surrounding the puncture; (*c*) ventral bone surface of larger costal (SNHM 1450/17): circular puncture with depressed fractures (white asterisk); (*e*) ventral bone surface of smaller costal (SNHM 1451): three shallow pits (white asterisks) and set of sub-parallel scores (white arrows); the latter could reflect distance of adjacent teeth in crocodylian jaw; (*f*) dorsal bone surface of larger hyo- or hypoplastron fragment (SNHM 1453/17): bisected mark (orientation indicated by white arrowheads) with depressed fractures (white asterisks) likely produced by unworn carinated tooth; (*g,h*) dorsal and ventral bone surface of smaller nuchal (SNHM 1449/17): bisected mark (orientation indicated by white arrowheads) completely piercing the bone. Note loss of internal cortical bone surrounding the puncture hole; (*i*) dorsal bone surface of smaller nuchal (SNHM 1449/17): bisected mark (orientation indicated by white arrowheads); (*j*) posterior bone surface of fused ischia (SNHM 1457/17): deep oblique furrows (white arrows); (*k*) dorsal bone surface of fused pubes (SNHM 1456/17): puncture with loss of internal cortical bone around it (in comparison with (*c*) and (*h*) impact likely occurred from the opposite side of the bone) and about nine short, deeper furrows (white arrow heads) and few longer shallow scores (white arrows); (*l*) ventral bone surface of fused pubes (SNHM 1456/17): same puncture as in (*k*) with depressed fractures (white asterisk) and deep furrow (white arrow); (*m*) dorsal bone surface of fused pubes (SNHM 1456/17): three hook scores (white arrows).

We identified several structures on the tortoise bones as crocodylian feeding traces (figure 4). Following the feeding trace identification used in Drumheller & Brochu [23], circular punctures with depressed fractures are found on the larger nuchal plate (SNHM 1448/17) and the fused pubes (SNHM 1456/17). The outline is somewhat irregular and chips of bone have flaked off the internal (i.e. visceral) surface of the tortoise shell bones. Bisected marks in the form of punctures or pits are found on the smaller nuchal (SNHM 1449/17) and on the larger hyo-/hypoplastron fragment (SNHM 1453/17), indicating that these traces might have been inflicted by unworn carinated teeth (comparable to similar feeding traces on mammal bones [72]). Again, the bisection pits are accompanied by flakes of internal compact bone. Circular pits that do not penetrate the shell bones completely are present on the larger nuchal plate (SNHM 1448/17) externally and internally, the smaller costal internally (SNHM 1451/17), the smaller hyo-/hypoplastron fragment externally (SNHM 1452/17), and on the small shell fragment (SNHM 1454/17) externally. Superficial scores (shallow parallel scratches, scores and J-shaped hook scores) are recognized only on the dorsal surface of the fused pubes (SNHM 1456/17). In addition, two deep oblique furrows are found on the posterior surface of the fused ischia (SNHM 1457/17). None of the shell or skeletal bones surrounding the feeding traces shows any signs of healing processes or callus formation.

## 4. Discussion

### 4.1. Crocodylians as the sole source for feeding traces

So far, there is no evidence for the presence of predatory mammals in the Pleistocene sediments on Aldabra, whereas crocodylian remains are well represented (e.g. [62,70]). Furthermore, the feeding traces encountered in the newly collected material do not hint at any mammalian dental features, but are all referable to the conical or carinated teeth typical of modern crocodylians [73] (see also crocodylian feeding traces on larger mammal bones [72]).

Crocodylian remains from the Pleistocene of Aldabra, specifically the Basin Cabri locality on Picard Island, and the Pointe Hodul locality of Grande Terre Island, have been described previously [58,62,74]. The latter were subsequently identified as belonging to a new crocodylid species, *Aldabrachampsus dilophus* Brochu, 2006 [62], based mostly on a very characteristic broadly convex crest on the squamosals, protruding posterolaterally as a set of ‘horns’, but the species is furthermore distinguishable ‘from all extant crocodylians on the basis of a dorsoventrally low premaxilla with highly vaulted palatal surface and anterodorsally oriented external naris and linear arrangement of premaxillary alveoli’ [62, p. 151]. Given the overall small size of the fragments of the holotype specimen BMNH R8795, as well as of the referred specimens, which were identified as belonging to a skeletally mature animal (or several individuals), the total body length was estimated to be approximately 2–2.5 m [62,74]. Based on the fragmentary nature of the finds, the phylogenetic position could not be fully resolved, but a close relationship either with other members of the genus *Crocodylus* or with osteolaemines such as *Voay* (‘*Crocodylus*’) *robustus* [75] from Madagascar, also a horned species (see [63,76] for descriptions of morphology), was suggested [62]. The question remains, however, whether a crocodylian of 2–2.5 m length would attack a fully grown giant tortoise with straight carapace lengths approaching or exceeding 1 m.

The newly recovered crocodylian material, on the other hand, is interpreted to belong to animals that were distinctly larger than previous estimates for *A. dilophus*. Comparing the size and dimensions of the new materials, such as the larger dentary fragment and the skull roof fragment, with the holotype material and referred specimens of *A. dilophus*, the former are not only larger but also more robust. This becomes apparent when the new specimens (and the right dentary and the left squamosal of the holotype of *A. dilophus*, BMNH R8795) are scaled and fitted to a similarly robust skull and associated lower jaw of an extant *C. niloticus* (ZM 100.302: ‘no data’ specimen; identification based on skull morphology; dorsal cranial length of 46 cm; figure 3). Thus, based on the new fossils, a reconstructed dorsal cranial skull length of 40–50 cm is feasible. Using these numbers in the allometric regressions of Webb & Messel [77] for *Crocodylus porosus*, the animal had a purported snout vent length of 140–175 cm, which corresponds to a total body length of approximately 290–370 cm. The new material could therefore belong either to an individual of *A. dilophus* distinctly larger than previously reported or to a different species. Both assumptions are equally possible based on the currently available fossil material. The presence of a different crocodylid species at Aldabra immigrated from elsewhere, however, is not unlikely, because crocodylids can survive in salt water (among others, [78–80]). *Crocodylus porosus* is well known to disperse across salt water [57,81,82] and indeed inhabited the granitic Seychelles until its extirpation in 1819 [58].

### 4.2. Scenarios explaining feeding traces

**Figure 5.**
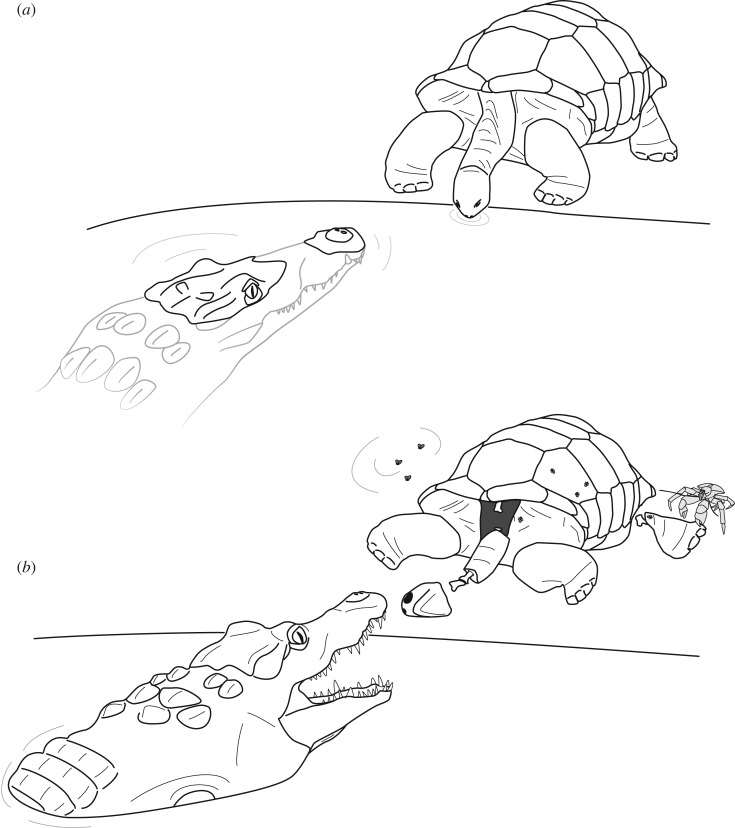
Possible Pleistocene trophic interaction scenarios including crocodylian and giant tortoise based on new fossil evidence. (*a*) Hunting crocodylian attracted by drinking tortoise. The attack likely occurred frontally or fronto-laterally where the head, neck and soft tissue parts of the anterior shell aperture are exposed; (*b*) decomposing tortoise carcass at breakdown stage 2 (putrid stage, with dipterans and ants [43]) attracting scavenging crocodylian and coconut crab. The spreading of the latter throughout the Indo-Pacific region has been proposed to have happened during the Pleistocene [83], and today this crab is one of the most active decomposition agents on Aldabra Atoll [43]. As in the previous scenario, the crocodylian is hypothesized to approach the carcass from the front, at the point of easiest access to the viscera.

Two scenarios are equally possible to explain the feeding traces on the giant tortoise bones (figure 5). In both cases, the tortoise bones were not ingested by the crocodylians, as can be deduced by their generally well-preserved state.

(1) Living giant tortoises were attacked by the crocodylians. This likely would have occurred as an ambush attack in a water hole, where the tortoise cannot easily spot the attacker. The feeding marks on the nuchals might support the notion that the crocodylian attacked the tortoise frontally or from an anterolateral direction when the tortoise came to drink, rather than from the lateral side, where the tortoise shell is highest. The absence of healing on the tortoise shells would indicate that the animal did not survive the encounter with the crocodylian.
(2) The crocodylians scavenged on tortoise carcasses. A recently deceased tortoise close to a waterhole could have attracted crocodylians which show opportunistic scavenging behaviour [57,84]. Similar to scenario 1, the shell apertures, especially the high anterior one, facilitate easier access to the viscera than the lateral flanks of the shell. With the tortoise already dead, no healing of the tortoise shell is to be expected either. Dead tortoises would also have been scavenged on by the smaller *Aldabrachampsus dilophus*, among other animals such as the coconut crab.

Besides the localized feeding traces on the shell and pelvic bones, the tortoise bones themselves are well preserved and appear to have separated by decay and not forcefully by shaking or inertial feeding [29,44,45].

In the case of the smaller Hungarian Late Cretaceous bothremydid turtle remains from Iharkút, differently arranged tooth row marks were proposed to be the result of inertial feeding as the turtle might have been moved around by the predator [10, p. 314]. We speculate that a giant tortoise of huge dimensions (size) as indicated by the recent fossil finds (figure 3*c*,*d*) would not have been easy to handle by a crocodylian predator/scavenger. As such, equidistant punctures and pits on the larger nuchal might indicate repeated biting with rearrangement of the crocodylian jaws on the shell, rather than vice versa.

According to Hu *et al.* [33], large to very large turtles or tortoises grow to such sizes that predators are basically unable to break apart their shells. Modern *A. gigantea*, and their relatives that lived during the Late Pleistocene, reach masses beyond 100 kg (reported up to 120–140 kg and 130–140 cm CCL for the largest males in the wild [85]), putting them well into the ‘size refuge’ *sensu* [33]. In those cases where the shell could thus not be cracked open, the predator/scavenger likely fed only on the exposed soft tissues of the neck and head or the limbs (compare also to [44]).

### 4.3. Ancient and modern ecosystems on Aldabra

In the recent past, the population of *A. gigantea* on Aldabra recovered from near extinction in the nineteenth century to a high of about 130 000 during the 1970s and was estimated at around 100 000 individuals by the end of the twentieth century [86,87]. There is no current population estimate available, but a recent analysis of data from a monitoring database showed that the population has been stable over the last 15 years [71]. The population details of *Aldabrachelys* tortoises on the atoll during the Late Pleistocene are currently not known, but this ancient ecosystem differed considerably to that of today, with crocodylians acting as apex predators. While the population details of the crocodylians are similarly unquantifiable, these animals nevertheless influenced the ecosystem structure by directly impacting the numbers of their giant living prey and also by lowering the persistence time of tortoise carcasses through scavenging. Today, the persistence time of those is much higher due to the absence of larger predators and decomposition is mainly achieved through invertebrate agents and weathering [43]. Modern crocodylians also have a good olfactory sense and using chemical cues allows them to detect deceased animals over large distances [57,88]. In this regard, the Pleistocene crocodylians likely also scavenged on carcasses that were not in or close to a pool of water. Late Pleistocene fossil material is abundant on Aldabra, and collections of additional material from the Cinq Cases region and elsewhere on the atoll could allow population-level morphological studies of the tortoises and crocodylians that would shed further light on the details of the extinct trophic relationship (e.g. prey size selection).

## 5. Conclusion

Recent collection efforts in the Late Pleistocene sediments on the coralline Aldabra Atoll yielded several bones of giant tortoises with bone surface structures identified as feeding traces (scores, pits, hooks and punctures) of crocodylians. From the same site, cranial and postcranial crocodylian remains were recovered that are interpreted herein as the cause of those feeding traces. The size of these remains is larger than previously collected crocodylian remains from Aldabra, indicating the hitherto unknown presence of a crocodylian with larger body size on the atoll (either belonging or not to the same taxon), which likely constituted an apex predator of the ancient ecosystem. This is the first report of direct trophic interaction in the fossil record of these vertebrate groups for the atoll.

## Data accessibility

All fossil specimens are stored in openly accessible, public repositories (see Institutional abbreviation section).
